# Does coffee consumption impact on heaviness of smoking?

**DOI:** 10.1111/add.13888

**Published:** 2017-07-12

**Authors:** Jennifer J. Ware, Julie‐Anne Tanner, Amy E. Taylor, Zhao Bin, Philip Haycock, Jack Bowden, Peter J. Rogers, George Davey Smith, Rachel F. Tyndale, Marcus R. Munafò

**Affiliations:** ^1^ MRC Integrative Epidemiology Unit (IEU) at the University of Bristol UK; ^2^ UK Centre for Tobacco and Alcohol Studies, School of Experimental Psychology University of Bristol UK; ^3^ School of Social and Community Medicine University of Bristol UK; ^4^ Campbell Family Mental Health Research Institute Centre for Addiction and Mental Health (CAMH) Toronto Canada; ^5^ Department of Pharmacology and Toxicology, and Psychiatry University of Toronto Canada; ^6^ School of Experimental Psychology University of Bristol UK; ^7^ MRC Biostatistics Unit Cambridge UK

**Keywords:** Causal inference, coffee, cigarette smoking, CYP2A6 metabolism, smoking heaviness, Mendelian randomisation

## Abstract

**Background and Aims:**

Coffee consumption and cigarette smoking are strongly associated, but whether this association is causal remains unclear. We sought to: (1) determine whether coffee consumption influences cigarette smoking causally, (2) estimate the magnitude of any association and (3) explore potential mechanisms.

**Design:**

We used Mendelian randomization (MR) analyses of observational data, using publicly available summarized data from the Tobacco and Genetics (TAG) consortium, individual‐level data from the UK Biobank and *in‐vitro* experiments of candidate compounds.

**Setting:**

The TAG consortium includes data from studies in several countries. The UK Biobank includes data from men and women recruited across England, Wales and Scotland.

**Participants:**

The TAG consortium provided data on *n* ≤ 38 181 participants. The UK Biobank provided data on 8072 participants.

**Measurements:**

In MR analyses, the exposure was coffee consumption (cups/day) and the outcome was heaviness of smoking (cigarettes/day). In our *in‐vitro* experiments we assessed the effect of caffeic acid, quercetin and p‐coumaric acid on the rate of nicotine metabolism in human liver microsomes and cDNA‐expressed human CYP2A6.

**Findings:**

Two‐sample MR analyses of TAG consortium data indicated that heavier coffee consumption might lead to reduced heaviness of smoking [beta = −1.49, 95% confidence interval (CI) = −2.88 to −0.09]. However, *in‐vitro* experiments found that the compounds investigated are unlikely to inhibit significantly the rate of nicotine metabolism following coffee consumption. Further MR analyses in UK Biobank found no evidence of a causal relationship between coffee consumption and heaviness of smoking (beta = 0.20, 95% CI = –1.72 to 2.12).

**Conclusions:**

Amount of coffee consumption is unlikely to have a major causal impact upon amount of cigarette smoking. If it does influence smoking, this is not likely to operate via effects of caffeic acid, quercetin or p‐coumaric acid on nicotine metabolism. The observational association between coffee consumption and cigarette smoking may be due to smoking impacting on coffee consumption or confounding.

## Introduction

Coffee consumption is associated with smoking 1, 2, 3. Observational studies show that coffee drinkers are more likely to be smokers than non‐consumers 2, 3 and smoke more heavily 1. This may arise via a number of possible mechanisms—constituents of coffee may have a pharmacological impact on the actions or metabolism of nicotine, or conditioning processes may lead to learned associations between the consumption of one and the other 1. Given the widespread use of coffee world‐wide, and the substantial health burden posed by cigarette smoking, determining the potential causal impact of coffee consumption on smoking behaviour is of clear public health importance. However, traditional observational epidemiological studies cannot determine direction of causality confidently, and cannot rule out the possibility of confounding by an unmeasured (or poorly measured) variable.

Mendelian randomization (MR) is a method which can be applied to observational data in order to: (a) provide evidence for the existence of causal relationships between variables and (b) estimate the magnitude of these relationships 4. It uses genetic variants that have been shown to be associated robustly with an exposure (e.g. coffee consumption) as proxies for the exposure. MR relies on the basic (but approximate) laws of Mendelian genetics: segregation and independent assortment. If these two laws hold, then, at a population level, genetic variants will not be associated with the confounding factors that can distort conventional observational studies. Furthermore, the gene variants with which individuals are born should not be altered by environmental factors, such as the outcome of interest, which removes the issue of reverse causality. This approach has been used to examine the causal impact of cigarette smoking on a variety of health outcomes 5, 6, 7. The identification of genetic variants associated with coffee consumption 8, 9, 10, 11, 12 presents the opportunity to use MR to examine the causal impact of coffee consumption on smoking.

Using conventional MR, the existence and magnitude of causal associations between an exposure and an outcome are determined by assessing the gene–exposure and gene–outcome relationships in the same data set or sample, where data on the genetic variant, exposure and outcome are available for all participants (i.e. one‐sample MR). However, it is also possible to use two independent data sets or samples to assess each of these relationships, one of which includes data on genotype and the exposure, and the other on genotype and the outcome. This approach is known as two‐sample MR 13, and is useful when it is hard to find study populations with data on the required genetic variant(s), exposure and outcome, particularly those of an adequate size for MR analyses (which typically require sample sizes in the 10s of 1000s). The two‐sample approach can also be applied to publicly available summary data from large‐scale genetic association studies 14. Here we applied a two‐sample MR approach, using publicly available data, to explore the existence of a causal effect of coffee consumption on smoking heaviness, and followed this up with *in‐vitro* experiments and replication in UK Biobank. We sought to: (1) determine whether coffee consumption causally influences cigarette smoking, (2) estimate the magnitude of any association and (3) explore potential mechanisms.

## Study 1

### Design

We performed two‐sample MR analyses, in which evidence for gene–exposure and gene–outcome associations were taken from different sources, using publicly available summarized data, in order to determine whether coffee consumption causally influences cigarette smoking, and estimate the magnitude of any association. This approach has been described elsewhere by Burgess and colleagues 14.

### Methods

We used summary‐level data from the European replication sample Coffee and Caffeine Genetics Consortium (CCGC) (*N* ≤ 30 062*)* for the gene–exposure association. The phenotype in this genome‐wide association study (GWAS) was cups of coffee consumed per day among consumers. Estimates for the associations of SNPs with coffee consumption were taken from analyses restricted to individuals of European ancestry. Full details of how this coffee consumption was assessed and control for population stratification in each contributing cohort are available in the supplementary material of the GWAS publication 8. Gene outcome associations were obtained from summary level data from the Tobacco and Genetics (TAG) consortium (cigarettes per day phenotype, *n* ≤ 38 181) and the cotinine consortium for the gene–outcome associations (*n* ≤ 4548) (see Supporting information).

Three specific combinations of single nucleotide polymorphisms (SNPs) that capture coffee consumption were used, corresponding to three models: (1) a set of eight independent (*r* ^2^ < 0.0006) SNPs (rs1260326, rs1481012, rs4410790, rs7800944, rs17685, rs6265, rs2470893, rs9902453), comprising variants which met the threshold for genome‐wide significance (log_10_ Bayes factor > 5.64) in the trans‐ethnic GWAS meta‐analysis of coffee consumption reported by the CCGC; (2) a moderate set of six SNPs (rs1260326, rs4410790, rs7800944, rs17685, rs2470893, rs9902453), which limited the full set to those variants which met the threshold of *P* < 0.05 in the CCGC stage 2 European sample GWAS; and (3) a conservative set of two SNPs (rs4410790, rs2470893), which limited the full set to variants in loci also identified in previous GWAS, in or near genes with a biological role in caffeine metabolism 8, 9, 10, 11, 12. The gene–exposure and gene–outcome associations were identified in the CCGC 8, TAG 15 and cotinine 16 consortia summary‐level data. Models 2 and 3 were included as sensitivity analyses, as recommended by Burgess and colleagues 14. These used variants with stronger evidence for association with coffee consumption (model 2) and those with the most specific phenotypical impact (model 3), enabling us to assess the possibility that our primary results (model 1) were attributable to genetic pleiotrophy (Table 1).

**Table 1:** Coffee consumption and smoking heaviness (cigarettes per day).

SNP	Gene	Model inclusions			Effect allele	Other allele	Association with coffee (cups per day)^a^		Association with smoking (cigarettes per day)^b^	
							Beta	Standard error	Beta	Standard error
		8‐SNP	6‐SNP	2‐SNP						
rs1260326	*GCKR*	Yes	Yes	No	C	T	0.03	0.01	−0.0821	0.0826
rs1481012	*ABCG2*	Yes	No	No	A	G	0.03	0.02	0.0297	0.1355
rs4410790	*AHR*	Yes	Yes	Yes	C	T	0.05	0.01	−0.0679	0.0906
rs7800944	*MLXIPL*	Yes	Yes	No	C	T	0.06	0.02	−0.0881	0.0973
rs17685	*POR*	Yes	Yes	No	A	G	0.05	0.01	0.1387	0.1377
rs6265	*BDNF*	Yes	No	No	C	T	0.03	0.01	0.0465	0.1045
rs2470893	*CYP1A1*	Yes	Yes	Yes	T	C	0.09	0.01	−0.2125	0.0964
rs9902453	*EFCAB5*	Yes	Yes	No	G	A	0.03	0.01	−0.0642	0.0817

For all three models, linkage disequilibrium (LD) pruning was performed: where pairs of variants were in LD, one of the variants was removed at random, ensuring total independence of all single nucleotide polymorphisms (SNPs) included in each model (*r* ^2^ < 0.0006 in all instances). The rs6968554 SNP, used in the 2 SNP model, was not included in the full 8 SNP model due to high LD with rs4410790 (*r* ^2^ = 0.99).

a Betas and standard errors from replication sample of Coffee and Caffeine Genetics Consortium (CCGC) genome‐wide association study (GWAS) meta‐analysis, *n* ≤ 30 062.

b Betas and standard errors from discovery sample of Tobacco and Genetics (TAG) GWAS meta‐analysis, *n* ≤ 38 181.

The causal change in smoking heaviness per unit increase in coffee consumption (i.e*.* per additional cup of coffee consumed per day) was estimated using the Wald ratio, with standard errors approximated by the delta method 17, 18. The SNP score is weighted according to associations with coffee consumption (measured as cups per day) in the CCGC GWAS (e.g. an allele which increases coffee consumption by 0.1 cups per day is given a value of 0.1). Therefore, the association with the outcome measure is expressed as the difference in outcome per additional cup of coffee consumed per day. Wald ratios were estimated for each SNP separately and combined by fixed‐effects meta‐analysis. This statistical approach has been described in full elsewhere 14. We also conducted a sensitivity analysis using the weighted median function approach 19. This approach is a way to test further the validity of a multi‐SNP instrument, as it generates a consistent estimate of causal effect even when up to 50% of the information in the analysis comes from SNPs that are invalid (e.g. subject to pleiotrophy). It therefore provides a simple means of assessing the robustness of the standard MR analysis to violations of MR assumptions (e.g. pleiotropy). All 95% CIs were calculated as 1.96 x SE. All statistical analyses were conducted using the R statistical software package (x64 version 3.0.1). Figures were generated using Stata version 11.

### Results

#### Smoking heaviness (cigarettes per day)

**Figure 1:**
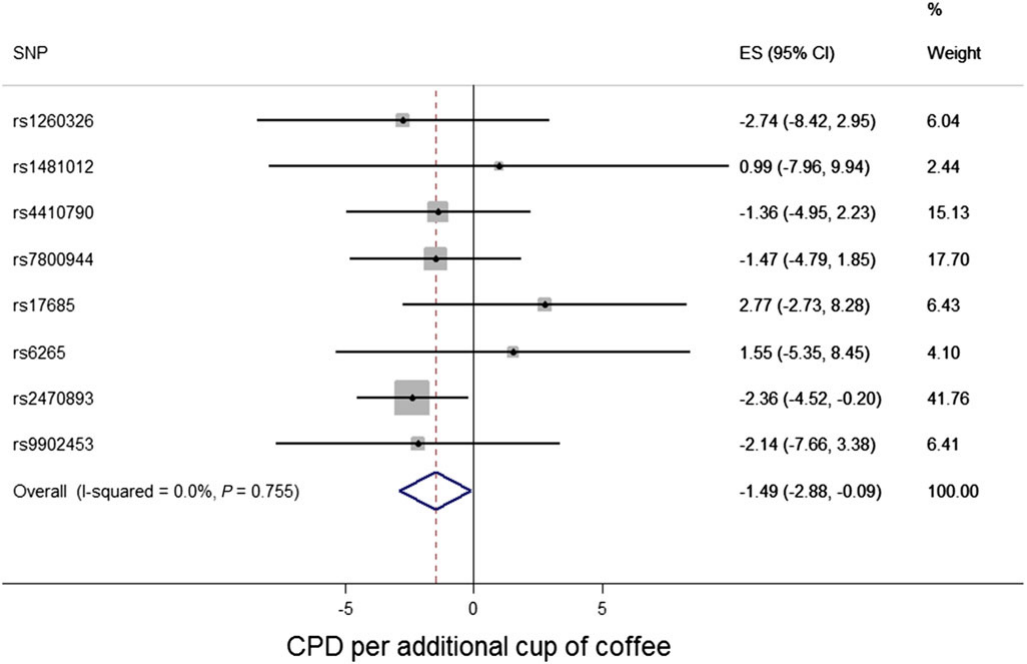
Coffee consumption and smoking heaviness forest plot (cigarettes per day). Effect size (ES) estimates reflect change in daily cigarette consumption per additional cup of coffee consumed per day. CI = confidence interval; CPD = cigarettes per day; SNP = single nucleotide polymorphism. [Colour figure can be viewed at wileyonlinelibrary.com]

There was evidence in support of a causal effect of coffee consumption on smoking heaviness. Each additional cup of coffee consumed per day corresponded to a decrease in daily cigarette consumption of approximately 1.5 cigarettes per day within a fixed‐effects framework (eight‐SNP model: beta = −1.49, 95% CI = –2.88 to −0.09, *P* = 0.037; six‐SNP model: beta = −1.69, 95% CI = –3.13 to −0.24, *P* = 0.022; two‐SNP model: beta = −2.09, 95% CI = –3.94 to −0.24, *P* = 0.027) (Table 1, Fig. 1). The weighted median function approach using all eight SNPs also generated a similar estimate (beta = −2.15, 95% CI = –3.73 to −0.07).

#### Smoking heaviness (cotinine levels)

**Figure 2:**
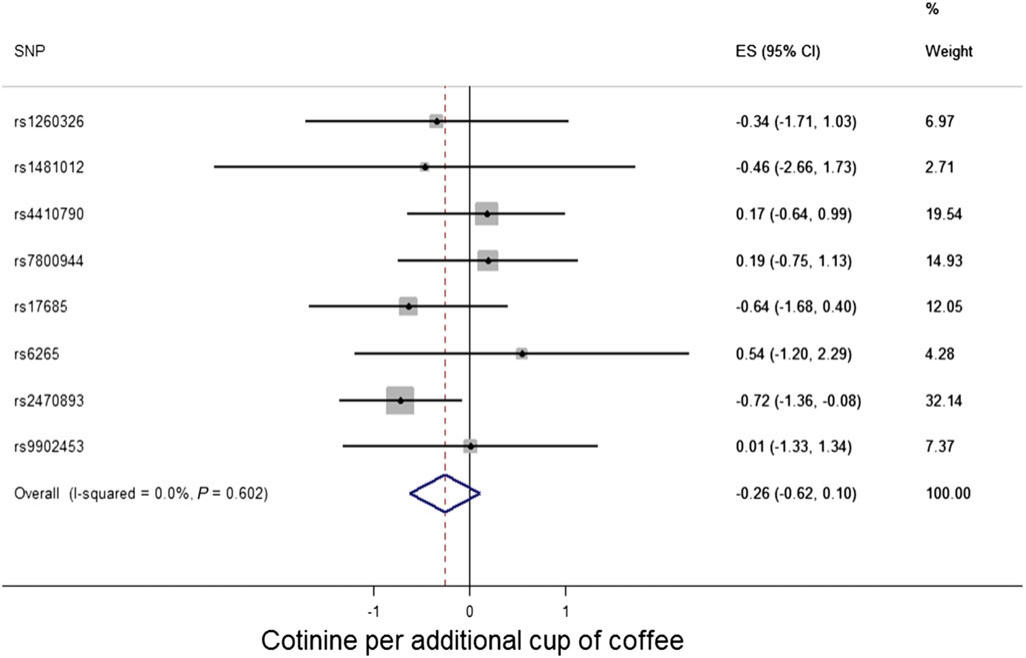
Coffee consumption and smoking heaviness forest plot (cotinine). Effect size (ES) estimates reflect standard deviation change in cotinine levels per additional cup of coffee consumed per day. CI = confidence interval. [Colour figure can be viewed at wileyonlinelibrary.com]

There was no clear evidence in support of a causal effect of coffee consumption on cotinine levels, although the direction of effect was consistent with that observed for daily cigarette consumption (i.e*.* a negative effect size estimate was observed) (eight‐SNP model: beta = −0.26, 95% CI = –0.62 to 0.10, *P* = 0.16; six‐SNP model: beta = −0.29, 95% CI = –0.66 to 0.09, *P* = 0.13; two‐SNP model: beta = −0.38, 95% CI = –0.88 to 0.12, *P* = 0.14) (Table 2, Fig. 2). Beta values refer to the standard deviation (SD) change in cotinine levels per cup of coffee consumed per day. For reference, a 0.26 SD decrease in cotinine level corresponds to ~46 ng/ml decrease in plasma/serum cotinine.

**Table 2:** Coffee consumption and smoking heaviness (cotinine).

SNP	Gene	Model inclusions			Effect allele	Other allele	Association with coffee (cups per day)^a^		Association with smoking (cotinine)^b^	
							Beta	Standard error	Beta	Standard error
		8‐SNP	6‐SNP	2‐SNP						
rs1260326	*GCKR*	Yes	Yes	No	C	T	0.03	0.01	−0.0102	0.0207
rs1481012	*ABCG2*	Yes	No	No	A	G	0.03	0.02	−0.0139	0.0323
rs4410790	*AHR*	Yes	Yes	Yes	C	T	0.05	0.01	0.0087	0.0208
rs7800944	*MLXIPL*	Yes	Yes	No	C	T	0.06	0.02	0.0114	0.0284
rs17685	*POR*	Yes	Yes	No	A	G	0.05	0.01	−0.0319	0.0258
rs6265	*BDNF*	Yes	No	No	C	T	0.03	0.01	0.0163	0.0262
rs2470893	*CYP1A1*	Yes	Yes	Yes	T	C	0.09	0.01	−0.0647	0.0284
rs9902453	*EFCAB5*	Yes	Yes	No	G	A	0.03	0.01	0.0002	0.0204

For all three models, linkage disequilibrium (LD) pruning was performed: where pairs of variants were in LD, one of the variants was removed at random, ensuring total independence of all single nucleotide polymorphisms (SNPs) included in each model (*r* ^2^ < 0.0006 in all instances). The rs6968554 SNP, used in the 2 SNP model, was not included in the full 8 SNP model due to high LD with rs4410790 (*r* ^2^ = 0.99).

a Betas and standard errors from replication sample of Coffee and Caffeine Genetics Consortium (CCGC) genome‐wide association study (GWAS) meta‐analysis, *n* ≤ 30 062.

b Betas and standard errors from discovery sample of cotinine GWAS meta‐analysis, *n* ≤ 4548.

### Discussion

These results suggested that coffee consumption may reduce heaviness of smoking, in contrast to evidence from observational studies. Cigarette consumption can decrease without cotinine levels being altered (e.g. if fewer cigarettes are smoked more intensively), so our results for cigarettes per day and cotinine are not necessarily inconsistent. Inhibition of CYP2A6‐mediated nicotine metabolism by constituents of coffee prolongs nicotine's clearance may be similar to the established impact of reduced nicotine metabolizer phenotype on reducing smoking in these smoking coffee consumers 20. It is known that smoking induces the enzyme cytochrome P450 1A2 (CYP1A2) (the main enzyme involved in caffeine metabolism), and therefore increases metabolism of caffeine 21, 22. It is also possible that constituents of coffee may have a pharmacological impact on the actions or metabolism of nicotine 1. Previous *in‐vitro* research has shown that caffeic acid and quercetin, compounds present in coffee, unrelated to caffeine, inhibit bovine cytochrome P450 2A6 (CYP2A6) enzyme activity 23. CYP2A6 plays a major role in nicotine metabolism, and is responsible for ~80% of the inactivation of nicotine to cotinine 24. A reduction in nicotine metabolism via inhibition of CYP2A6 activity would result in longer‐lasting circulating levels of nicotine per unit intake, thus extending its window of pharmacological effect and leading to a reduction in smoking. Previous human experimental studies have focused on manipulation of caffeine dose, rather than coffee dose, and show little or no evidence of a relationship between caffeine dose and cigarette consumption 25, 26, but this is not inconsistent with the mechanism we propose.

It is important to note that the most strongly associated coffee‐related SNPs identified to date (in or near *AHR* and *CYP1A2*) increase coffee consumption by increasing caffeine metabolism, leading to a decrease in circulating caffeine levels. This has been shown in a GWAS of blood metabolites and of caffeine metabolites 27, 28. Caffeine is metabolized primarily by the enzyme CYP1A2 29, but does not inhibit nicotine pharmacokinetics *in‐vivo* nor does it alter cigarette consumption 25, 30. For example, when given caffeine at a high dose (12 mg/kg/day, approximately 800 mg per person, relative to average daily consumption of 200 mg) there was no effect on nicotine or cotinine plasma levels or on nicotine intake, cigarettes smoked or grams of tobacco burned 23; therefore, caffeine was not investigated further as a potential CYP2A6 inhibitor. Instead we explored the inhibitory potential of three alternative compounds present in coffee, caffeic acid, quercetin and p‐coumaric acid on human nicotine metabolism 31. In contrast, caffeic acid and quercetin reduce the rate of metabolism of the CYP2A6 substrate, coumarin, in bovine liver microsomes and were thus investigated as potential inhibitors of human nicotine metabolism 23.

## Study 2

### Design

Using 8‐methoxypsoralen as a positive inhibitory control 32, we assessed the effect of caffeic acid, quercetin and p‐coumaric acid on the rate of nicotine metabolism in human liver microsomes and cDNA‐expressed human CYP2A6 in order to explore potential mechanisms for the results observed in study 1.

### Methods

(−)‐Nicotine hydrogen tartrate, (−)‐cotinine, caffeic acid, quercetin, p‐coumaric acid and 8‐methoxypsoralen were purchased from Sigma‐Aldrich (St Louis, MO, USA). CYP2A6 supersomes were purchased from BD Gentest (Woburn, MA, USA). The custom‐made internal standard, 5‐methylcotinine, was purchased from Toronto Research Chemicals (Toronto, ON, Canada). Human liver microsome preparation and *in‐vitro* inhibition of nicotine metabolism were performed as described previously 32, 33; further details can be found in the Supporting information. The inhibitory potency of each inhibitory test compound was determined by incubating human liver microsomes or CYP2A6 supersomes with nicotine and various concentrations of each compound under linear conditions for nicotine metabolism. Positive control experiments were also conducted using the established CYP2A6 inhibitor 8‐methoxypsoralen 32. We assessed the velocity of cotinine formation (V, nmol/mg protein/min for microsomes, nmol/pmol CYP2A6/min for supersomes) and the inhibitor constant (K_i_) for each test compound; K_i_ values were estimated using Dixon plots, while Cornish–Bowden plots were used to infer the type of inhibition (competitive, non‐competitive, uncompetitive or mixed). The relative impact of inhibitor pre‐incubation versus no pre‐incubation on the rate of cotinine formation was used to investigate whether or not the compounds had the potential to act as irreversible inhibitors. Analyses were performed using GraphPad Prism version 6.0.

### Results

#### Inhibition of in‐vitro nicotine metabolism by caffeic acid, quercetin and p‐coumaric acid

**Figure 3:**
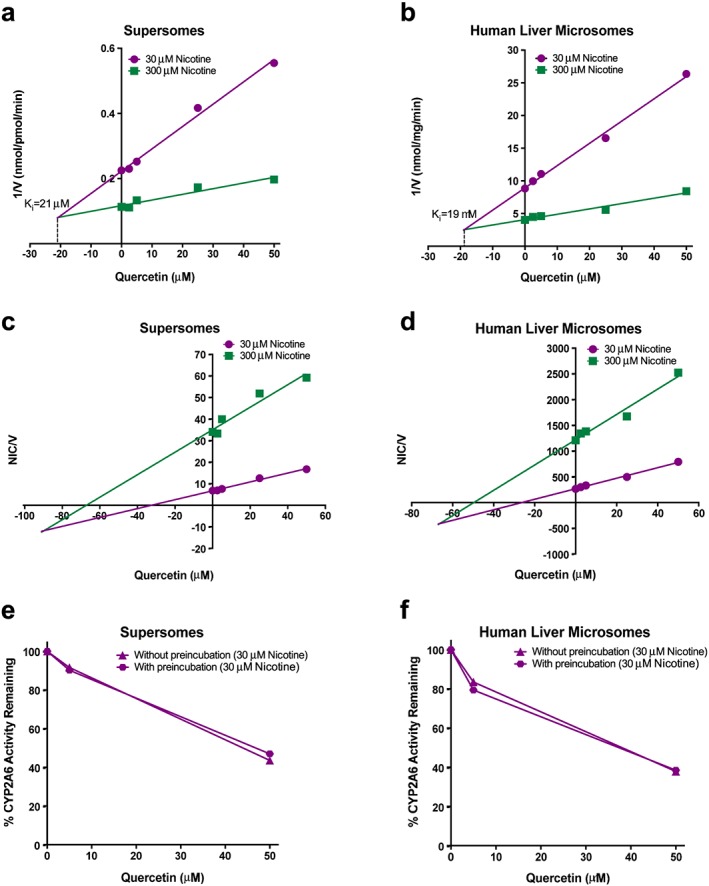
Inhibition of cytochrome P450 2A6 (CYP2A6) activity by quercetin. Dixon plots of the inhibition of nicotine metabolism by CYP2A6 in the presence of increasing concentrations of quercetin in (a) human CYP2A6 supersomes and (b) human liver microsomes (without pre‐incubation). Cornish–Bowden plots of nicotine (μM)/velocity (nmol/min/mg) with increasing concentrations of quercetin in (c) human CYP2A6 supersomes and (d) human liver microsomes (without pre‐incubation). Comparison of CYP2A6 inhibition by quercetin with and without pre‐incubation, at increasing inhibitor concentrations in (e) human CYP2A6 supersomes and (f) human liver microsomes. Velocity (V), supersomes: nmol cotinine/min/pmol CYP2A6, microsomes: nmol cotinine/min/mg. [Colour figure can be viewed at wileyonlinelibrary.com]

All three test coffee compounds inhibited CYP2A6 activity, reducing the rate of cotinine formation from nicotine. In human liver microsomes and CYP2A6 supersomes quercetin (Fig. 3) had the lowest inhibitory constant (lowest K_i_), and thus the highest affinity and greatest inhibition against cotinine formation (microsomes 19 μM, supersomes 21 μM), followed by caffeic acid (microsomes 152 μM, supersomes 156 μM) and p‐coumaric acid (microsomes 247 μM, supersomes 243 μM) (Supporting information, Figs S1, S2a,b). All three compounds exhibited substantially lowered inhibitor potency compared to our positive control, 8‐methoxypsoralen (microsomes 0.18 μM, supersomes 0.20 μM; Supporting information, Fig. S3a,b). Caffeic acid and p‐coumaric acid demonstrated characteristics of competitive inhibitors (Supporting information, Figs S1, S2c,d), while quercetin appeared to act as a mixed inhibitor (Fig. 3c,d). 8‐Methoxypsoralen, an established *in‐vitro* mechanism‐based inhibitor for CYP2A6, showed the expected mixed inhibition 34, which is typical for these *in‐vitro* tests for a mechanism‐based inhibitor. The K_i_ and type of inhibition for each compound has been summarized in Supporting information, Table S2.

#### Effect of pre‐incubation with inhibitor on in‐vitro nicotine metabolism

We pre‐incubated human liver microsomes and CYP2A6 supersomes with each inhibitor prior to administration of nicotine to assess if they might act as irreversible inhibitors. Mechanism‐based inhibition is such that the compound is activated metabolically by the enzyme, and subsequently the product binds irreversibly 35; we would expect to observe increased inhibition of cotinine formation with inhibitor pre‐treatment relative to standard inhibitor treatment for a mechanism‐based inhibitor. There was no difference in the percentage of CYP2A6 activity (cotinine formation from nicotine) remaining after treatment with increasing concentrations of the test compounds (caffeic acid, quercetin and p‐coumaric acid) between the inhibitor pre‐treatment and non‐pre‐treatment conditions (Fig. 3, Supporting information, Figs S1, S2e,f), suggesting that these compounds do not act as irreversible inhibitors. Pre‐treatment with 8‐methoxypsoralen was associated with a greater decrease in the percentage of CYP2A6 activity remaining relative to the non‐pre‐treatment group (Supporting information, Figure S3e,f), consistent with its known function as an *in‐vitro* mechanism‐based inhibitor of CYP2A6 34.

### Discussion

Our results indicated that three test compounds present in coffee—caffeic acid, quercetin and p‐coumaric acid—each act as inhibitors of cotinine formation from nicotine. The test inhibitors are present at low amounts in coffee; therefore, to be capable of inhibiting nicotine metabolism sufficiently enough to reduce smoking, they would have to be either (1) very potent or (2) a mechanism‐based‐inhibitor. The potency of inhibition varied between each compound, and was several magnitudes lower than that of 8‐methoxypsoralen, our positive inhibitory control. The type of inhibition also differed between compounds. Competitive inhibition, which was demonstrated by caffeic acid and p‐coumaric acid towards cotinine formation, is characterized by reversible binding of the inhibitor to the substrate‐binding site, with the potential for this inhibition to be overcome by high concentrations of the substrate (i.e*.* nicotine) 36. Quercetin exhibited characteristics of a mixed inhibitor, suggesting that this compound binds to the vacant, or already substrate‐bound, enzyme with greater affinity for one state or the other 37. 8‐Methoxypsoralen was the only compound to act as a mechanism‐based inhibitor, in agreement with the literature 34. 8‐Methoxypsoralen inhibits nicotine metabolism *in‐vitro* and *in‐vivo* significantly and decreases smoking, and therefore was a suitable positive control for this study 38, 39.

Based on these findings, we can predict roughly whether the amount of each compound present in one cup of coffee is adequate to substantially inhibit nicotine metabolism and reduce smoking. The amount of total caffeic acid, quercetin and p‐coumaric acid per cup of coffee is between 6 and 175 mg, 0.005 mg and 0.14 mg, respectively 40, 41, 42. Considering the relatively low quantities of quercetin and p‐coumaric acid present in coffee, and the inhibitory potencies of both compounds, it is unlikely that either compound is responsible for substantial inhibition of nicotine metabolism following coffee consumption. However, caffeic acid intake from coffee is much higher, due probably to the presence of free caffeic acid in coffee and the formation of caffeic acid in the body as a metabolic product of chlorogenic acid, which is found at amounts ranging from 10 to 350 mg per cup of coffee 41, 42, 43, 44. Caffeic acid and caffeine are actually present at similar amounts in coffee, with one cup of coffee containing approximately 100 mg of each compound 41. The average plasma levels of caffeine among smokers has been assessed after consumption of increasing amounts of coffee 45, and we can use these data to roughly estimate caffeic acid plasma concentrations (see Supporting information). A plasma caffeic acid concentration of 1.11 mg/l (6.16 μM), corresponding to a consumption of two to three cups of coffee, would result in 3.8% greater inhibition of nicotine metabolism relative to no caffeic acid. When smokers consume three to four cups of coffee per day, their plasma caffeic acid concentration is predicted to be 1.28 mg/l (7.12 μM), resulting in a 4.4% inhibition of nicotine metabolism. With each additional cup of coffee consumed, the inhibition of nicotine metabolism increases by < 1%. Based on the shorter half‐life and lower levels of caffeic acid compared to caffeine, our assumptions overestimate the potential effects of caffeic acid; based on these over‐assumptions, there was still little support that caffeic acid from coffee consumption was probably able to inhibit nicotine metabolism sufficiently to result in a decrease of 1.5 cigarettes per day with the consumption of each additional cup of coffee.

Our findings therefore suggest that caffeic acid, quercetin and p‐coumaric acid inhibit CYP2A6 enzymatic activity, but with relatively low inhibitory potency. This, along with the amount of each compound present in coffee, suggests that these compounds do not inhibit the rate of nicotine metabolism following coffee consumption substantially, and that these coffee constituents are unlikely explanations for the findings in study 1. We therefore explored whether the results of study 1 could be replicated in a large cohort study, the UK Biobank.

## Study 3

### Design

We performed MR analyses using individual data from the UK Biobank study, in order to replicate the findings rom study 1.

### Methods

The UK Biobank (www.ukbiobank.ac.uk) recruited more than 500 000 men and women (aged 37–73 years) between 2006 and 2010 46. Participants attended one of the 21 assessment centres in England, Wales and Scotland, where they provided information on demographic, life‐style factors and medical history through interviews and questionnaires and had physical measurements and blood, urine and saliva samples taken. The full protocol for the study is available online: www.ukbiobank.ac.uk/wp‐content/uploads/2011/11/UK‐Biobank‐Protocol.pdf. The UK Biobank study was approved by the North West Multi‐Centre Research Ethics Committee and all participants provided written informed consent to participate in the UK Biobank study.

Genotyping of the eight coffee‐related variants (rs4410790, rs2470893, rs1260326, rs1481012, rs7800944, rs9902453, rs17685, rs6265) was conducted in an initial sample of 152 249 individuals. Full details of the genotyping are provided in Supporting information. The analysis sample was restricted to unrelated individuals, based on a threshold of 0.05 estimated from genetic kinships, and to individuals of European genetic ancestry using principal components analyses (PCA). This resulted in a sample of 114 963 with genetic data. Two weighted genetic risk scores were calculated; one using all eight SNPs and the other using just the SNPs in *AHR* and *CYP1A1* (rs4410790 and rs2470893). SNPs were weighted using beta coefficients from the replication stage of the CCGC GWAS (Supporting information, Table S3).

Individuals were asked: ‘How many cups of coffee do you drink each day?’. Answers were provided on a continuous scale. For the purposes of this analysis, no distinction was made between caffeinated and decaffeinated coffee consumption. Current regular smokers were asked about number of cigarettes consumed per day; answers were provided on a continuous scale.

Analyses were conducted in Stata version 14 and R version 3.0.1. The analysis sample was restricted to current daily smokers, who reported consuming at least some coffee. We assessed observational associations between coffee consumption and daily cigarette consumption using linear regression. To assess the validity of the instrument, we tested the association between genetic risk scores for coffee consumption and self‐reported coffee consumption and a range of possible confounders (age, sex, income, educational attainment, deprivation index). We conducted the Mendelian randomization analysis in the same way as the two‐sample analysis, by calculating Wald ratios for each SNP separately and combining using inverse variance weighted fixed‐effects meta‐analysis. We also performed a median weighted regression as a sensitivity analysis 19. Regressions were adjusted for age, sex and the 15 principal genetic components provided by UK Biobank. Robust standard errors were used to account for non‐normality of residuals. All 95% CIs were calculated as 1.96 x SE.

### Results

Characteristics of the study sample are shown in Supporting information, Table S4. Median coffee consumption was three cups per day [interquartile range (IQR) = 2–5] and median cigarette consumption was 15 cigarettes per day (IQR = 15–20). Among the 8072 current daily smokers who reported consuming coffee, each additional cup of coffee consumed per day was associated with smoking 0.45 additional cigarettes per day (95% CI = 0.39–0.52). Both the eight‐SNP and two‐SNP genetic risk scores for coffee were associated with coffee consumption; however, only the eight‐SNP genetic risk score had an *F*‐statistic > 10 (*F* = 20.8), indicating that it was likely to be a sufficiently strong instrument for the analysis. Therefore, only the results using the eight‐SNP score are presented here. The average effects per coffee consumption increasing allele were 0.08 additional cups per day (95% CI = 0.04–0.11) for the eight‐SNP score. Neither the eight‐SNP nor the two‐SNP scores were associated with potential confounders (Supporting information, Table S4).

**Figure 4:**
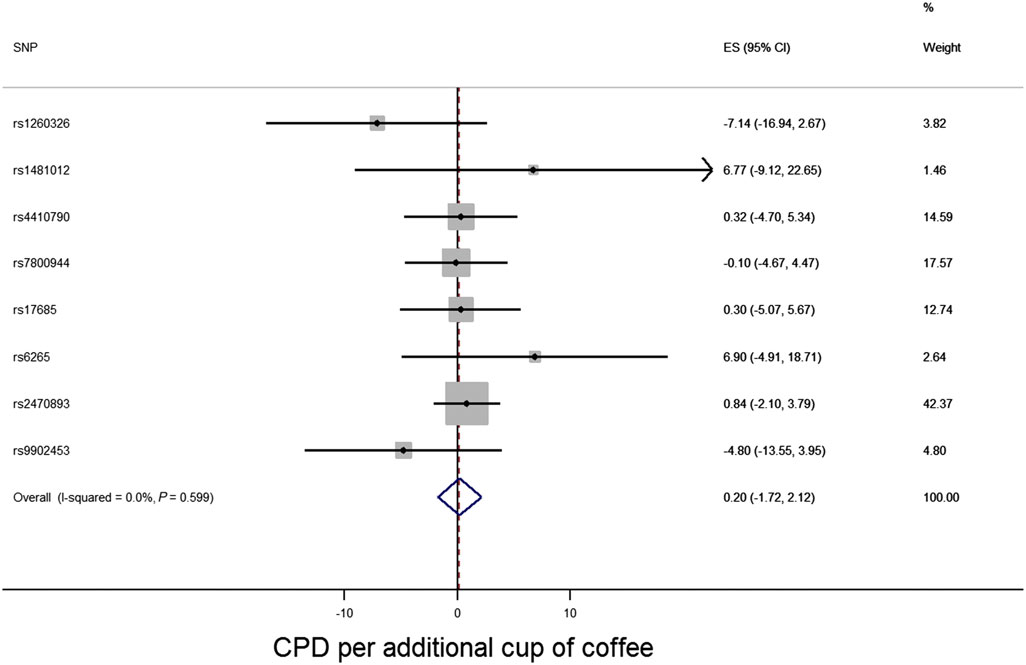
Coffee consumption and smoking heaviness forest plot (cigarettes per day) in UK Biobank. Effect size (ES) estimates reflect change in cigarettes per day per additional cup of coffee consumed per day; CI = confidence interval; SNP = single nucleotide polymorphism. [Colour figure can be viewed at wileyonlinelibrary.com]

The associations of the coffee‐related SNPs with cigarette consumption in UK Biobank are shown in Table 3. When Wald ratios were combined in a fixed‐effects meta‐analysis, there was no clear evidence for a causal effect of coffee consumption on daily cigarette consumption (beta per additional cup of coffee consumed = 0.20 additional cigarettes per day, 95% CI = –1.72 to 2.12, *P* = 0.84) using the eight‐SNP score (Fig. 4). This was consistent with the estimate from median weighted regression (0.38, 95% CI = –1.83 to 2.59, *P* = 0.74). As the first release of UK Biobank GWAS data includes a case–control study which selected individuals on the basis of lung function (high, medium and low) and smoking status (never smoking and heavy smoking) (the UK Bileve study 47), we also repeated this analysis excluding these individuals (*n* = 4751) and results were similar (data not shown).

**Table 3:** Coffee consumption and smoking heaviness (cigarettes per day) in UK Biobank.

SNP	Gene	Model inclusions		Effect allele	Other allele	Association with coffee (cups per day)^a^		Association with smoking (cigarettes per day)^b^	
						Beta	Standard error	Beta	Standard error
		8‐SNP	2‐SNP						
rs1260326	*GCKR*	Yes	No	C	T	0.03	0.01	−0.214	0.132
rs1481012	*ABCG2*	Yes	No	A	G	0.03	0.02	0.203	0.202
rs4410790	*AHR*	Yes	Yes	C	T	0.05	0.01	0.016	0.128
rs7800944	*MLXIPL*	Yes	No	C	T	0.06	0.02	−0.006	0.140
rs17685	*POR*	Yes	No	A	G	0.05	0.01	0.015	0.137
rs6265	*BDNF*	Yes	No	C	T	0.03	0.01	0.207	0.167
rs2470893	*CYP1A1*	Yes	Yes	T	C	0.09	0.01	0.076	0.135
rs9902453	*EFCAB5*	Yes	No	G	A	0.03	0.01	−0.144	0.125

a Betas and standard errors from replication sample of CCGC genome‐wide association study (GWAS) meta‐analysis, *n* ≤ 30 062.

b Betas and standard errors from UK Biobank, *n* = 8072.

SNP = single nucleotide polymorphism.

### Discussion

These results failed to support the observation in study 1, that consumption may reduce heaviness of smoking. Indeed, the point estimates were in the direction of an increase in heaviness of smoking. This, together with the results of study 2, suggests that coffee consumption does not causally decrease heaviness of smoking. Some caution must be exercised, as the confidence intervals were wide and included effects that might be considered of public health relevance (either an increase or decrease in heaviness of smoking equivalent to one cigarette per day per additional cup of coffee consumed per day, based on the 95% confidence interval around our point estimate), but the point estimate was in the opposite direction to that observed in study 1.

## General Discussion

Taken together, our results suggest that coffee consumption is unlikely to have a major causal impact on cigarette smoking. If it does influence smoking, this is not likely to operate via effects of caffeic acid, quercetin or p‐coumaric acid on nicotine metabolism. The observational association between coffee consumption and cigarette smoking may be due to smoking impacting upon coffee consumption, or confounding. It is likely that at least some of the positive observational association between coffee consumption and cigarette smoking is due to the impact of smoking upon coffee intake. There is evidence from Mendelian randomization analysis, using a genetic variant that determines heaviness of smoking 48, that heavier smoking increases coffee consumption causally 49.

There are some limitations to our approach that should be considered. First, pleiotropy is a potential problem in Mendelian randomization analyses. We therefore performed analyses using all eight SNPs, but also restricting to the two SNPs that are involved directly in caffeine metabolism. We also conducted a sensitivity analysis using the weighted median function approach, which generates a consistent estimate of causal effect even when up to 50% of the information in the analysis comes from SNPs that are invalid (i.e. pleiotropic). These produced similar results, suggesting that the results are less likely to be due to pleiotrophic effects of a single SNP, or that if pleiotrophy is present, it is not influencing our results substantially. Secondly, our Mendelian randomization may have lacked power to detect small effects. In the UK Biobank sample, we estimate that we had between 61 and 89% power to detect an association of −1.5 cigarettes per day per additional cup of coffee consumed (based on SDs of 2 and 8.5 for coffee and cigarettes per day, respectively, alpha of 0.05 and the genetic risk score explaining between 0.5 and 1% of variance in coffee consumed). Thirdly, we used only a limited number of SNPs in our Mendelian randomization analyses; a polygenic risk score including a larger number of SNPs might have provided greater power by capturing more variance in coffee consumption. Unfortunately, the full results of the GWAS of coffee consumption that we used are not publicly available, so we were able to use only the variants that reached genome‐wide significance and were reported. Fourthly, we did not distinguish between caffeinated and decaffeinated coffee consumption. The CCGC GWAS phenotype was ‘predominantly regular‐type’ of coffee consumption (which is likely to include some decaffeinated consumption), although they also ran separate analyses looking specifically at decaffeinated coffee consumption. They found evidence that SNPs near *AHR* also increased decaffeinated coffee consumption, due probably to a continuation of coffee consumption behaviour in individuals who have switched from caffeinated to decaffeinated consumption. Given that the genetic variants are likely to determine both types of consumption and it is difficult to distinguish accurately between the two, we do not think that further stratification of these analyses into decaffeinated and caffeinated consumers is likely to be informative. Fifthly, in our *in‐vitro* experiments, we did not investigate the effects of coffee itself. Caffeine has already been tested and has not been shown to inhibit CYP2A6 activity or influence cigarette consumption 25, 30. The compounds we investigated were all putative inhibitors based on the literature, their structures and the amounts in coffee 23. Kinetic assessments on compound mixtures such as coffee are possible, but would provide little indication of the type of inhibition and the duration of action (i.e. mechanism‐based inhibitors may elicit a prolonged inhibitory effect relative to competitive inhibitors).

What these results highlight is the need to be wary of potential chance findings, in particular where statistical evidence is modest (as in study 1). They also illustrate the need to triangulate potentially important findings with both mechanistic studies that interrogate putative biological pathways (study 2) and direct replication studies (study 3), where possible using methods that support stronger causal inference 50. Given the growing concerns that many published biomedical research findings may be false 51, and that the reproducibility of much research is consequently lower than is desirable 52, we believe that approaches similar to that we have adopted here will be increasingly important.

### Declaration of interests

M.R.M. reports grants from Pfizer and other research support from GlaxoSmithKline, outside the submitted work. A.E.T. reports grants from Pfizer, outside the submitted work. P.J.R. reports grants from GlaxoSmithKline, outside the submitted work. R.F.T. reports consultancy for Apotex, outside the submitted work. All other authors report no competing interests.

## Supporting information




**Table S1** Phenotypes associated with included single nucleotide polymorphisms (SNPs) as reported in the genome‐wide association study (GWAS) Catalog and/or PubMed.
**Table S2** The inhibitory potency of caffeic acid, quercetin, p‐coumaric acid and 8‐methoxypsoralen (positive control) on nicotine metabolism by CYP2A6.
**Table S3** Genetic variants used as proxies for smoking heaviness and tea and coffee consumption in unrelated European individuals in UK Biobank.
**Table S4** Characteristics of the UK Biobank analysis sample (n = 8072).
**Figure S1** Inhibition of cytochrome P450 1A2 (CYP2A6) activity by caffeic acid.
**Figure S2** Inhibition of cytochrome P450 1A2 (CYP2A6) activity by p‐coumaric acid.
**Figure S3** Inhibition of cytochrome P450 1A2 (CYP2A6) activity by 8‐methoxypsoralen.
