# Supplementary material for: Does coffee consumption impact on heaviness of smoking?
Source: Addiction. 2017 Jul 12;112(10):1842–53. doi: 10.1111/add.13888 (PMC5600104; doi:10.1111/add.13888)
Supplement: Supplementary file 1 — Table S1 Phenotypes associated with included single nucleotide polymorphisms (SNPs) as reported in the genome‐wide association study (GWAS) Catalog and/or PubMed. Table S2 The inhibitory potency of caffeic acid, quercetin, p‐coumaric acid and 8‐methoxypsoralen (positive control) on nicotine metabolism by CYP2A6. Table S3 Genetic variants used as proxies for smoking heaviness and tea and coffee consumption in unrelated European individuals in UK Biobank. Table S4 Characteristics of the UK Biobank analysis sample (n = 8072). Figure S1 Inhibition of cytochrome P450 1A2 (CYP2A6) activity by caffeic acid. Figure S2 Inhibition of cytochrome P450 1A2 (CYP2A6) activity by p‐coumaric acid. Figure S3 Inhibition of cytochrome P450 1A2 (CYP2A6) activity by 8‐methoxypsoralen. [file ADD-112-1842-s001.docx]

**Supplementary Material**

**Study One**

**Study samples**

For gene-exposure (i.e., gene-coffee consumption) associations we used data from a genome-wide association study (GWAS) of coffee consumption published by the Coffee and Caffeine Genetics Consortium (CCGC) [^1^](#_ENREF_1), comprised of 48 studies using samples of European and African American ancestry. Detailed information on study design and populations can be found in the supplementary material accompanying the original study. [^1^](#_ENREF_1) For gene-outcome associations we used data from GWASs of smoking behaviour phenotypes published by the Tobacco and Genetics (TAG) consortium [^2^](#_ENREF_2), accessed via the Psychiatric Genomics Consortium website (<http://www.med.unc.edu/pgc/downloads>). The specific smoking phenotype GWAS dataset used was daily cigarette consumption (N = 38,181). Descriptive characteristics of the 16 studies participating in the TAG consortium can be found in the original study [^2^](#_ENREF_2). Data from a GWAS meta-analysis of cotinine levels (the primary metabolite of nicotine) in daily smokers of European ancestry were also used to further explore the causal impact of coffee consumption on heaviness of smoking (N = 4,548) [^3^](#_ENREF_3). Gene-exposure effect size estimates and standard errors were limited to those observed in Stage 2 of the CCGC GWAS analyses (N = 30,062, all European ancestry) in order to avoid sample overlap with the TAG consortium data, since two-sample MR assumes that the data sources for the gene-exposure and gene-outcome associations are independent [^4^](#_ENREF_4). Ethics approval for this study was not required.

**Study Two**

Microsomal membranes were prepared from human livers, and were stored at -80°C in 1.15% KCl, as previously described [^5^](#_ENREF_5). Human liver tissue was generously provided by Dr. T. Inaba (University of Toronto, Toronto, ON, Canada), from the K-series liver bank, as previously described [^6^](#_ENREF_6). During membrane preparation, cytosolic fractions were collected and used as a source of aldehyde oxidase in all *in vitro* nicotine metabolism assays. Protein concentrations were quantified using Bradford reagent according to the manufacturer’s protocol (Bio-Rad Laboratories Ltd.).

**In vitro inhibition of nicotine metabolism.**

The inhibitory potency of each inhibitory test compound was determined by incubating human liver microsomes or CYP2A6 supersomes with nicotine and various concentrations of each compound. Inhibitor concentrations were experimentally determined based on their ability to decrease cotinine formation from nicotine, under conditions designed to determine the K_i_ and mechanism of action for each potential inhibitor. Positive control experiments were also conducted using the established CYP2A6 inhibitor 8-methoxypsoralen [^7^](#_ENREF_7). Using human liver microsomes, linear conditions of nicotine metabolism were established for assay conditions of 0.5 mg/ml protein for an incubation time of 20 min. Using CYP2A6 supersomes, linear conditions of nicotine metabolism were established for assay conditions of 10 pmol P450/ml (containing P450 reductase and cytochrome b5) for an incubation time of 15 min. Incubation mixtures contained 1 mM NADPH, 1 mg/ml cytosol, 50 mM Tris-HCl buffer (pH 7.4), and vehicle or inhibitor in 2% DMSO. Incubations were performed at 37°C in a final volume of 0.5 ml. We established a K_m_ of approximately 30 μM for cotinine formation from nicotine in pooled human liver microsomes; this value is within the wide range of K_m_ values (10-160 μM) previously reported for cotinine formation in human liver microsomes [^6^](#_ENREF_6). Nicotine was used at concentrations of 30 and 300 μM (K_m_ and ~10X K_m_, respectively); inhibitor concentrations used were: caffeic acid 0, 2.5, 25, 50, 500 μM, quercetin 0, 2.5, 5, 25, 50 μM, p-coumaric 0, 2.5, 25, 50, 500 μM, and 8-methoxypsoralen 0, 0.05, 0.5 μM. For incubations with a pre-incubation step, reactions containing inhibitor were initiated by pre-warming the mixture for 2 min at 37°C before the addition of NADPH, followed by a pre-incubation of 15 min at 37°C. This was then followed by the standard incubation procedure in which nicotine was added and the reaction mixture was incubated for 15 min (supersomes) or 20 min (microsomes) at 37°C. The reactions were terminated with a final concentration of 4% (v/v) Na_2_CO_3_ and 70 μg internal standard (5-methylcotinine) was added. Samples were extracted and analyzed by high performance liquid chromatography as previously described [^5^](#_ENREF_5). Limits of quantification for nicotine and cotinine were 5 ng/ml and 12.5 ng/ml, respectively.

**Estimation of caffeic acid plasma concentrations**

de Leon and colleagues [^8^](#_ENREF_8) showed that the average caffeine intake among smokers was approximately 3 mg/kg/day for a 70 kg person, which corresponds to the consumption of 2-3 cups of coffee per day, and a resulting plasma caffeine concentration of 1.11 mg/l (5.72 μM). If we assume that the amount of caffeine and caffeic acid are similar per cup of coffee and they have similar pharmacokinetic properties, this would suggest that smokers’ average daily caffeic acid intake is also 3 mg/kg/day, and they have a plasma caffeic acid concentration of 1.11 mg/l (6.16 μM). Using the Michaelis-Menten equation for competitive inhibition, we can estimate the degree of inhibition by caffeic acid on nicotine metabolism.

V=(Vmax*[S])/(Km*(1+ [I]/Ki)+[S])

V is the velocity of CYP2A6 mediated nicotine metabolism, Vmax and Km are kinetic parameters of nicotine metabolism by CYP2A6, [S] is substrate (nicotine) concentration and is equal to 30 ng/ml (or 0.2 μM), [I] is inhibitor (caffeic acid) concentration, and Ki refers to the inhibitory potency of caffeic acid toward nicotine metabolism by CYP2A6.

**Study three**

**Genotyping**

DNA was extracted from blood samples using the Promega Maxwell 16 Blood DNA Purification Kit (AS1010), which uses magnetic bead technology to purify the DNA. An initial sample of 152,249 individuals were genotyped for 641,018 SNPs, some using the Affymetrix UK BiLEVE Axiom array and some using the Affymetrix UK Biobank Axiom array. These two arrays are very similar, with an overlap of around 95%. Imputation was conducted using the 1000 genomes Phase 3 and UK10k reference panels. After quality control, just over 73 million SNPs were available for analysis.

**Supplementary Table S1. Phenotypes associated with included SNPs as reported in the GWAS Catalog and/or PubMed.**

| **SNP** | **Chr** | **Gene** | **Model inclusions** | | | **Phenotype** |
| --- | --- | --- | --- | --- | --- | --- |
|  |  |  | **8-SNP** | **6-SNP** | **2-SNP** |  |
| rs1260326 | 2 | *GCKR* | Yes | Yes | No | Blood metabolite levels (mannose; alanine; S-HDL-P)  Blood metabolite ratios (glucose/mannose; alanine/glutamine)  Cholesterol (total)  Triglycerides  Glycemic traits  Hypertriglyceridemia  Urate levels  Lipoprotein-associated phospholipase A2 mass  Serum albumin level  Serum protein level (total)  Platelet counts  Liver enzyme levels (gamma-glutamyl transferase)  C-reactive protein  Chronic kidney disease  Two-hour glucose levels  Lipid metabolism phenotypes (TG; VLDL)  Liver fat content  Gallbladder disease  Gout  Plasma viscosity  Non-alcoholic fatty liver disease  Glomerular filtration rate  Insulin and insulin resistance  Dyslipidaemia |
| rs1481012 | 4 | *ABCG2* | Yes | No | No | Gout  Response to statin therapy (LDL-C) |
| rs4410790 | 7 | *AHR* | Yes | Yes | No | Caffeine consumption |
| rs6968554^a^ | 7 | *AHR* | No | No | Yes | Blood caffeine levels |
| rs7800944 | 7 | *MLXIPL* | Yes | Yes | No | Triglycerides |
| rs17685 | 7 | *POR* | Yes | Yes | No | None |
| rs6265 | 11 | *BDNF* | Yes | No | No | Smoking initiation  BMI  Weight  Multiple candidate gene associations, including neurocognitive phenotypes, neuropsychiatric phenotypes (including addiction related phenotypes), and other disease phenotypes. |
| rs2470893 | 15 | *CYP1A1* | Yes | Yes | Yes | Coffee consumption  Caffeine consumption  Breast cancer risk (African Americans)  Polychlorinated biphenyl 118 levels |
| rs9902453 | 17 | *EFCAB5* | Yes | Yes | No | None |

^a^ rs6968554 was not included in the 8-SNP model due to high LD with rs4410790 (r^2^ = 0.99). Chr = chromosome.

**Supplementary Table S2. The inhibitory potency of caffeic acid, quercetin, p-coumaric acid, and 8-methoxypsoralen (positive control) on nicotine metabolism by CYP2A6.**

| **Compound** | **Enzyme source** | **K_i_ (μM)** | **Type of Inhibition** |
| --- | --- | --- | --- |
| **Caffeic acid** | CYP2A6 supersomes | 156 | Competitive |
|  | Human liver microsomes | 152 |  |
| **Quercetin** | CYP2A6 supersomes | 21 | Mixed, but not mechanism-based |
|  | Human liver microsomes | 19 |  |
| **p-Coumaric acid** | CYP2A6 supersomes | 243 | Competitive |
|  | Human liver microsomes | 247 |  |
| **8-Methoxypsoralen** | CYP2A6 supersomes | 0.18 | Mixed, mechanism-based |
|  | Human liver microsomes | 0.20 |  |

Estimated K_i_ is displayed for each compound as determined from varying concentrations of each compound incubated with CYP2A6 supersomes or human liver microsomes at two concentrations of nicotine (30 μM and 300 μM) *without* preincubation.

**Supplementary Table S3. Genetic variants used as proxies for smoking heaviness and tea and coffee consumption in unrelated European individuals in UK Biobank.**

| **SNP number** | **Gene** | **N** | **Effect allele** | **Effect allele frequency** | **HWE p-value** | **Directly genotyped or imputed** | **Imputation accuracy** | **Beta value for genetic risk score^1^** |
| --- | --- | --- | --- | --- | --- | --- | --- | --- |
| rs4410790 | *AHR* | 114,321 | C | 0.63 | 0.39 | Directly genotyped | - | 0.14 |
| rs2470893 | *CYP1A1* | 114,321 | T | 0.33 | 0.75 | Directly genotyped | - | 0.12 |
| rs1260326 | *GCKR* | 114,321 | C | 0.61 | 0.25 | Directly genotyped | - | 0.04 |
| rs1481012 | *ABCG2* | 112,762 | A | 0.89 | 0.48 | Imputed | 0.9845 | 0.06 |
| rs7800944 | *MLXIPL* | 113,427 | C | 0.29 | 0.56 | Imputed | 0.9954 | 0.05 |
| rs9902453 | *EFCAB5* | 113,897 | G | 0.45 | 0.08 | Imputed | 0.99784 | 0.04 |
| rs17685 | *POR* | 114,321 | A | 0.28 | 0.16 | Directly genotyped | - | 0.07 |
| rs6265 | *BDNF* | 114,321 | C | 0.81 | 0.84 | Directly genotyped | - | 0.05 |

1. Effect size of association with coffee (cups per day) from stage 1 GWAS of Coffee and Caffeine Genetics Consortium

**Supplementary Table S4. Characteristics of the UK Biobank analysis sample (N = 8,072).**

|  | Total sample | Association with 2 SNP genetic risk score  (Beta/OR, 95% CI)^1^ | P-value | Association with 8 SNP genetic risk score  (Beta/OR, 95% CI)^1^ | P-value |
| --- | --- | --- | --- | --- | --- |
| Age: Mean (SD, range) | 54.8 (8.0, 40 to 70) | -0.02 (-0.20, 0.17) | 0.87 | 0.02 (-0.07, 0.12) | 0.62 |
| Sex (N, % male) | 3,829 (47.4%) | 1.00 (0.96, 1.05) | 0.92 | 1.00 (0.97, 1.02) | 0.71 |
| Degree/professional qualifications (N, %) | 2,486 (31.3%) | 0.98 (0.93, 1.03) | 0.49 | 0.98 (0.96, 1.01) | 0.20 |
| Townsend deprivation index: Mean (SD, range) | 0.13 (3.49, -6.25 to 10.55) | 0.02 (-0.06, 0.10) | 0.66 | 0.02 (-0.02, 0.06) | 0.28 |
| Income ≥£52,000 (N, %) | 994 (14.2%) | 1.02 (0.95, 1.09) | 0.61 | 1.00 (0.96, 1.04) | 0.99 |

Missing data: Qualifications: N = 87, Townsend deprivation: N =13, Income: 1,074.

1. Beta from linear regression for continuous variables and OR from logistic regression for binary variables. Associations are the average effect per coffee consumption increasing allele.

**Supplementary Figure S1. Inhibition of CYP2A6 activity by caffeic acid.**


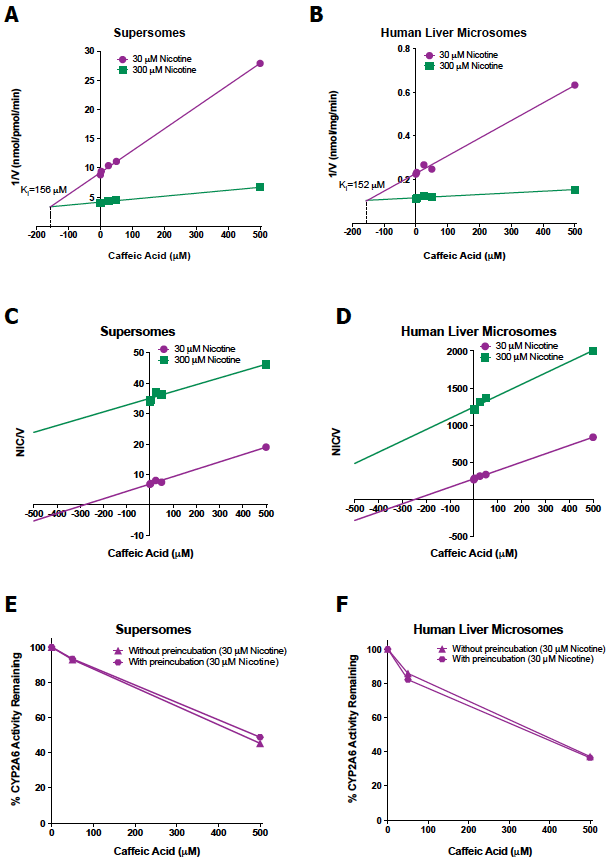


Dixon plots of the inhibition of cotinine formation by CYP2A6 in the presence of increasing concentrations of caffeic acid in (A) human CYP2A6 supersomes and (B) human liver microsomes (without preincubation). Cornish-Bowden plots of nicotine (μM)/velocity (nmol/min/mg) with increasing concentrations of caffeic acid in (C) human CYP2A6 supersomes and (D) human liver microsomes (without preincubation). Comparison of CYP2A6 inhibition by caffeic acid with and without preincubation at increasing inhibitor concentrations in (E) human CYP2A6 supersomes and (F) human liver microsomes. Velocity (V), supersomes: nmol cotinine/min/pmol CYP2A6, microsomes: nmol cotinine/min/mg.

**Supplementary Figure S2. Inhibition of CYP2A6 activity by p-coumaric acid.**


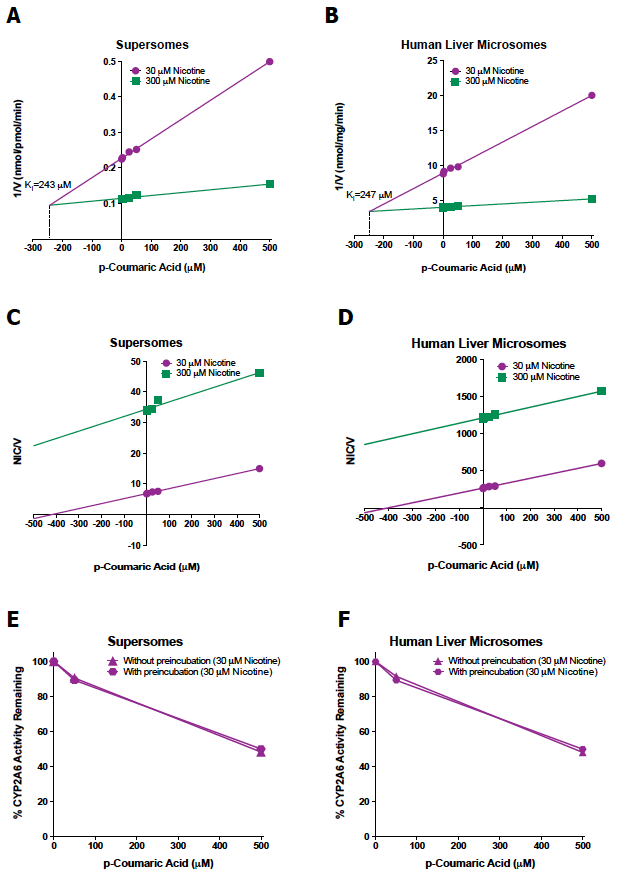


Dixon plots of the inhibition of nicotine metabolism by CYP2A6 in the presence of increasing concentrations of p-coumaric acid in (A) human CYP2A6 supersomes and (B) human liver microsomes (without preincubation). Cornish-Bowden plots of nicotine (μM)/velocity (nmol/min/mg) with increasing concentrations of p-coumaric acid in (C) human CYP2A6 supersomes and (D) human liver microsomes (without preincubation). Comparison of CYP2A6 inhibition by p-coumaric acid with and without preincubation, at increasing inhibitor concentrations in (E) human CYP2A6 supersomes and (F) human liver microsomes. Velocity (V), supersomes: nmol cotinine/min/pmol CYP2A6, microsomes: nmol cotinine/min/mg.

**Supplementary Figure S3. Inhibition of CYP2A6 activity by 8-methoxypsoralen.**


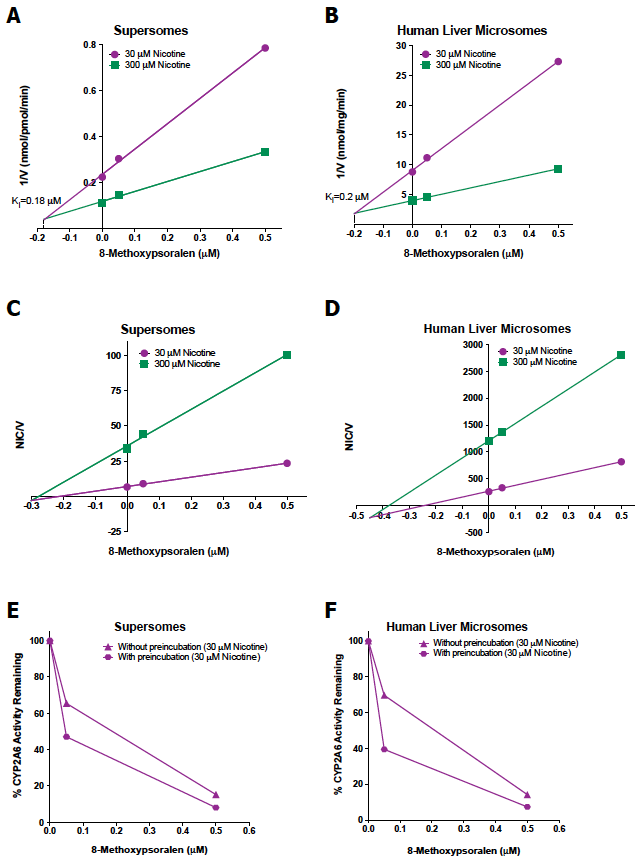


Dixon plots of the inhibition of nicotine metabolism by CYP2A6 in the presence of increasing concentrations of 8-methoxypsoralen in (A) human CYP2A6 supersomes and (B) human liver microsomes (without preincubation). Cornish-Bowden plots of nicotine (μM)/velocity (nmol/min/mg) with increasing concentrations of 8-methoxypsoralen in (C) human CYP2A6 supersomes and (D) human liver microsomes (without preincubation). Comparison of CYP2A6 inhibition by 8-methoxypsoralen with and without preincubation, at increasing inhibitor concentrations in (E) human CYP2A6 supersomes and (F) human liver microsomes. Velocity (V), supersomes: nmol cotinine/min/pmol CYP2A6, microsomes: nmol cotinine/min/mg.

**References**

1. The-Coffee-and-Caffeine-Genetics-Consortium, Cornelis MC, Byrne EM, et al. Genome-wide meta-analysis identifies six novel loci associated with habitual coffee consumption. *Molecular psychiatry.* 2014.

2. Tobacco, Genetics C. Genome-wide meta-analyses identify multiple loci associated with smoking behavior. *Nature genetics.* 2010;42(5):441-447.

3. Ware JJ, Chen X, Vink J, et al. Genome-wide meta-analysis of cotinine levels in cigarette smokers identifies locus at 4q13.2. *PNAS.* under review.

4. Burgess S, Butterworth A, Thompson SG. Mendelian randomization analysis with multiple genetic variants using summarized data. *Genetic epidemiology.* 2013;37(7):658-665.

5. Siu EC, Wildenauer DB, Tyndale RF. Nicotine self-administration in mice is associated with rates of nicotine inactivation by CYP2A5. *Psychopharmacology.* 2006;184(3-4):401-408.

6. Messina ES, Tyndale RF, Sellers EM. A major role for CYP2A6 in nicotine C-oxidation by human liver microsomes. *The Journal of pharmacology and experimental therapeutics.* 1997;282(3):1608-1614.

7. Zhang W, Kilicarslan T, Tyndale RF, Sellers EM. Evaluation of methoxsalen, tranylcypromine, and tryptamine as specific and selective CYP2A6 inhibitors in vitro. *Drug Metab Dispos.* 2001;29(6):897-902.

8. de Leon J, Diaz FJ, Rogers T, et al. A pilot study of plasma caffeine concentrations in a US sample of smoker and nonsmoker volunteers. *Prog Neuropsychopharmacol Biol Psychiatry.* 2003;27(1):165-171.
